# Nature Experiences of Older People for Active Ageing: An Interdisciplinary Approach to the Co-Design of Community Gardens

**DOI:** 10.3389/fpsyg.2021.702525

**Published:** 2021-09-27

**Authors:** Marco Boffi, Linda Pola, Natalia Fumagalli, Elisabetta Fermani, Giulio Senes, Paolo Inghilleri

**Affiliations:** ^1^Department of Cultural Heritage and Environment, University of Milan, Milan, Italy; ^2^Department of Agricultural and Environmental Sciences, University of Milan, Milan, Italy

**Keywords:** citizen engagement, attention restoration, biophilic design, focus group, participatory design

## Abstract

In Western countries, the concepts of healthy ageing and active ageing, that is, concepts that favour health, participation, and security to increase the quality of life of older people, have become key issues. Hence, the effective design of public green spaces in urban areas is crucial, as visiting these areas encourages social relations and interactions in natural, healthy contexts. Consistent with these perspectives, existing landscape design criteria emphasise the importance of considering not only the functional and aesthetic elements, but also the ecosystemic and biophilic relationships between people and the environment, producing positive effects for both. To maximise the impact of such design criteria, proper engagement strategies are desirable, both to assign an active role to older people themselves and to inform the fine-tuning of the design process according to the specific needs of the local population. This study presents an interdisciplinary co-design method that encourages and informs a biophilic approach by describing the experiences of people in natural environments, actual and designed, through the application of attention restoration theory (ART). The case study was developed through six focus group meetings with older people for the co-design of a restorative area in a community garden in the Ortica district in Milan (Italy). Results show how the main needs expressed by participants can be classified into the ART factors of “compatibility” (a multifunctional garden), “fascination” (sense of contact with nature), and “being away” (metaphorical escape from nursing homes). The garden designed includes biophilic principles that respond to such needs, and specific links with designed elements were identified. For example, “being away” (e.g., isolation from daily routine and visual occlusion of the surroundings) and “compatibility” (e.g., pergola and aesthetic value) are the factors that include the elements that more satisfactorily answer previous needs. “Fascination” also includes many positive aspects, allowing space for improvements (e.g., more water elements, interaction with animals). Implications of the method are discussed, including the importance of subjective experience in informing design, the use of different psychological constructs to describe it, and the methodological alternatives for psychological assessment.

## Introduction

An increasingly ageing population is a challenge for most of the European countries. Such a demographic change requires appropriate measures to help older people remain healthy and active, with these measures being consistent with the concepts of healthy ageing and active ageing that have been adopted in many countries to define appropriate guidelines. Healthy ageing refers to a “holistic perspective that includes subjective experiences and meanings, functional definitions emphasising autonomy, participation, and well-being” (Sixsmith et al., 2014, p. 1). Active ageing can be defined as “the process of optimising opportunities for health, participation, and security to enhance the quality of life as people age” (WHO, 2002 p. 12). As is recommended in the global age-friendly cities guide published by the WHO (2007), one way to ensure that our cities and communities meet the needs of the ageing population is to involve them in co-designing processes, which can be particularly fruitful in the case of nature-based solutions (Mahmoud and Morello, 2021). The relevance of natural elements to human health is sustained by a wide and growing body of research in the field of healthcare for older adults, showing that interactions with the urban green environment have a wide range of positive health outcomes and benefits. Spending time in natural surroundings encourages physical activity and engagement, increases energy, fights depression, boosts memory, improves physical health, reduces stresses, provides restorative experiences, and encourages social relations (Kaplan, 1995; Berman et al., 2008; Pearson and Craig, 2014; Capaldi et al., 2015; Lawton et al., 2017). This scenario emphasises the importance of properly designing green public spaces in urban areas, taking into account both the physical features of the environment and the subjective factors that are of importance to sensitive populations. In fact, in cities of today, the satisfactory experience of nature requires deliberate design and development decisions (Kellert, 2016). In this field, at the intersection between built and natural environments, two emerging design methods, biophilic design and biophilic urbanism, represent an inter-scalar approach to increasing connectivity to the natural environment. Biophilic design attempts to achieve the beneficial effects of contact with nature in the modern built environment by working, for example, at the architectural scale. The goal of biophilic urbanism is to reduce the contemporary urban disconnect from nature, making the experience of the natural world an integral part of ordinary city life; it aims to ameliorate the urban environment at the district or municipal scale. Rather than focusing on a single building, landscape, or human use, biophilic urbanism encompasses a vast array of ecological systems and human activities. They both define criteria and strategies to provide experiences of nature in urban areas that evoke positive physiological and psychological responses (Eid et al., 2021).

It is worth noting that, although a great emphasis is traditionally placed on the “organic or naturalistic dimension” (Kellert, 2008, p. 5) of the biophilic approach, by referring to forms that recall the human predisposition toward nature, this concept also encompasses a “place-based or vernacular dimension” (Kellert, 2008, p. 6), which involves a relationship with local cultural aspects. In such perspectives, the interdisciplinary exchange between the social sciences and design sciences is much needed today, which is in line with the concept of cities as artefacts that can be properly interpreted only by referring to both their spatial features and the social dynamics taking place there (Romice et al., 2016; Inghilleri, 2021). Although such perspectives are producing some results in the field of research and related experimental initiatives, the social sciences still play a limited role in design processes, which occur in everyday contexts. This not only diminishes the overall quality of design projects, but also becomes an obstacle to the full actualization of the positive potential of some technical solutions. For an effective interdisciplinary co-design approach, we can consider urban design as a recursive process that can be divided into five main design phases (analyze, plan, design, assess, and communicate) that cross six key actions (observe and interpret, measure and compare, model and simulate, strategize, design, and communicate) (Piga, 2017). According to such a scheme, the contribution of social sciences can occur during the entire design process, offering theories and models to observe and interpret the interactions between people and their environment and then providing tools for measuring and comparing that interaction consistently with the chosen theories. If such aspects are those traditionally faced by scholars in the field of environmental psychology, a further contribution to the participatory and communicative aspects of the process can come from community psychology. From this point of view, the social sciences can support designers and planners from the very beginning to the end of the process. However, such an influence is bidirectional, as the design sciences can inform about the needs emerging in specific contexts, contributing to the creation of integrated perspectives and tools that can more effectively build knowledge about the people–environment interaction. To maximise the impact of design criteria, proper engagement strategies are desirable, both to assign an active role to older people and to inform the fine-tuning of the design process according to the specific needs of local populations.

Overall, the goal of the current study is to comprehend how the key features of the experiences of older people, in their relationship with nature, affect their evaluation of biophilic environments. The understanding of this relationship can be achieved by integrating traditional top-down biophilic design criteria with bottom-up co-design data and by expanding the investigation area from the single site, typical of biophilic design, to the broader context, as suggested by biophilic urbanism. As no consolidated assessment tool has been developed in the field of biophilia, we borrowed the interpretative categories of the experiences in nature from attention restoration theory (ART) (Kaplan and Kaplan, 1989), a well-established model that has provided many quantitative instruments in the field of environmental psychology. From a theoretical perspective, the goal is to emphasise the points of contact between biophilic design and ART, with a strong link with actual designed physical elements. We aim to reconnect the early phases of the design practise (i.e., analyse, plan, and design; see Piga, 2017) with the existing principles of ART. From the methodological perspective, the goal is to develop a method that can be practically included in the design process. We aim to provide a qualitative tool to inform co-design activities with a theory-driven interpretation of the experiences of both citizens in nature and their reactions to design project proposals. The case study was developed in the Ortica district in Milan to redesign a portion of its community garden named “San Faustino,” creating a healing garden.

In the following section Design criteria for a restorative garden, we summarise the biophilic design criteria that can be considered when designing a community garden, with a specific focus on the needs of older people. We illustrate the ART (Kaplan and Kaplan, 1989), which is an effective conceptual and empirical bridge between design and psychology. In section Focus groups for citizens engagement, we reflect on the implications of adopting focus groups as a method for co-design, subsequently proposing an analysis approach that includes ART both for the observation of needs and for design project assessments. In section Method, we present the method, in general, and its main steps. In section Results, we illustrate the results obtained from each step. In section Discussion and conclusion, we discuss the implications of the case study and reflect on the future development of the method.

## Design Criteria for a Restorative Garden

Biologist Wilson (1984) asserted that humans have an innate predisposition, which he called biophilia, to connect with other forms of life; specifically, an innate and genetically determined affinity of human beings with the natural world. From an evolutionary standpoint, biophilia has promoted the successful adaptation to environmental conditions across hundreds of millennia in which humans have lived in close association with nature. “Biophilic rules persist from generation to generation, atrophied and fitfully manifested in the artificial new environments into which technology has catapulted humanity” (Wilson, 1993, p. 32). According to Letourneau (2013), present-day manifestations of biophilia in humans include the quickness and decisiveness with which humans learn particular things about certain kinds of plants and animals (Wilson, 1984); the attraction of modern humans to open spaces with lush vegetation, scattered trees, and conspicuous water features; the aversion of modern humans toward closed spaces (Heerwagen and Orians, 1993; Ulrich, 1993); the preference of modern humans for viewing natural landscapes over urban scenes (Ulrich, 1993); the stress-reducing and restorative effects of visiting, or even just viewing non-threatening, natural settings or water features, on modern humans (Berto et al., 2018) the therapeutic effects of contact with nature on modern humans (Ulrich, 1993); the enhancement of creativity, mental discipline, and higher-level cognitive functioning in modern humans associated with an exposure to nature (Ulrich, 1993; Faaber-Taylor et al., 1998; Bassi et al., 2018).

The evolutionary theory proposed by Wilson recognised the human life support function that nature plays in providing ecosystem services (Wang et al., 2021). It has oriented architectural and urban planning toward biophilic design, an extension of biophilia that tends to include natural systems and processes in buildings and constructed landscapes in order to provide human beings with their much-needed exposure to nature. This has led scholars and practitioners to reflect on the environmental qualities (e.g., light, colours, shapes, materials, and vegetation) that can have a positive impact on human physiology and psychology in order to design spaces capable of improving the experiences of different peoples.

Much of the literature available about biophilic design concerns architecture, but this design approach also finds a place in landscape architecture (Davidson, 2013); for biophilic design to be truly effective, it should extend beyond buildings into “life between buildings” (Moore and Marcus, 2008) and integrate ecology in a sustainable landscape design. Hence, biophilic urbanism offers a key contribution, as it develops biophilia at the urban scale. Bringing in biophilia at the urban scale implies the need to focus on the experience in the environment and to facilitate the relationship with nature through urban planning and design tools. Therefore, urban planning strategies and the design of urban spaces can foster direct (e.g., green infrastructures, parks, and green roofs) or indirect contact with nature (e.g., natural materials and green buildings) through the application of natural models and processes to the built spaces (Totaforti, 2020).

Biophilia, biophilic design, and biophilic urbanism offer an integrated and inter-scalar approach to fostering a renovated consciousness of our relationship with nature. They emphasise the importance of the world beyond ourselves in fostering our well-being, highlighting the notion of a relationship with the environment, in general, and with nature, in particular. From a design perspective, this must not be conceived as a mere decorative intervention, but rather as a systemic approach to landscape conception (Kellert, 2016).

The increasing attention to the therapeutic and regenerative effects that contact with nature has on humans has now opened numerous lines of research on the use of gardens in the health and care sectors and on a specific design approach named “healing garden design.” The formal design of gardens, seen as offshoots of internal environments, where the built prevails over the naturalistic component, does not exploit the regenerative and therapeutic potential that these spaces can offer. Frost (1992) speaks of “do-not-touch environments,” referring to those types of green spaces with a predominantly aesthetic function, as they are unable to involve and stimulate those for whom these spaces are intended and can inhibit interaction. The design of a healing/restorative garden is the combination of two conceptual components: the healing/restorative process and the space in which this process is supported (Marcus and Barnes, 1999). This suggests how the quality of both the environment and the experiences they offer can affect the healing process.

One important interaction between individuals and their environment is the restoration of our attention, our energy, and ourselves by experiencing or viewing nature (Clay, 2001). An urban lifestyle, from a psychological perspective, imposes a high demand on our cognitive systems: even if relaxing settings and activities may provide restorative opportunities, nature seems to be particularly useful for this purpose (Kaplan and Berman, 2010). “Restorative environments” refer to those natural places that favour a shift toward more positively toned emotional states and positive changes in physiological activity levels, cognitive functioning, and behaviour (Kaplan and Kaplan, 1989). They not only permit, but also promote restoration, thus enabling a faster, more complete recovery of depleted resources than environments that are relatively free of demands but which lack positive features (Hartig, 2004). It is an effect of the relationship between a user and setting without any therapeutic program or defined therapeutic activities (Haller, 2004). Research into the restorative benefits of contact with nature has generally looked at three main areas of contact: active, less active, and passive. Physical rehabilitation and engagement in horticultural therapy are examples of “active” interactions with the garden, while sitting in the garden, observing plants and animals, and listening to nature sounds are examples of “less-active” modes of interaction. However, the garden can also be experienced “passively” by viewing it through a window from the inside (Stigsdotter et al., 2011).

Most of the research projects related to restorative environments derive from the biophilia hypothesis and ART. Both have an evolutionary approach, maintaining that we are adapted through human evolution to function well in natural environments, although some natural environments will better serve restoration than other kinds of environments (Stigsdotter et al., 2011). Human tendencies to love and take care of nature are affected by attention, i.e. the ability to focus on natural stimuli effortlessly, actually to be fascinated by nature (Kaplan, 1995), and empathy, i.e. to feel connected emotionally to the various life forms, and to participate in their condition. Attention restoration theory (Kaplan and Kaplan, 1989) suggests that the ability to concentrate can be restored by exposure to natural environments and describes four different factors, the combination of which encourages “involuntary” or “indirect attention” and enables our “voluntary” or “directed” attention capacities to recover and restore ourselves (Kaplan, 1995). The Kaplans have established that, in order to be restorative, a place must have the following features (Kaplan and Kaplan, 1989): fascination, which is achieved through a setting that easily engages attention, thereby allowing fatigued attention to rest; being away, which is provided by a setting that is either physically or conceptually different from everyday settings of an individual; extent, which is provided by a setting that is complex enough to engage the mind and promote exploration; compatibility, which is achieved when the design of a setting supports the intended use by the occupant.

In the planning field, to help the restorative process and to support the needs of users, the design has to follow specific criteria that result from different disciplines: landscape architecture, medical sciences, ecopsychology, and landscape ecology. The design criteria can be summarised as follows (Fumagalli et al., 2020):

Prosthetic environment: one which compensates for cognitive deficits and positively influences the functional status and behaviour of older people whose ability to interact with their surroundings declines beyond their 80th year. It has to guarantee the optimal functioning of individuals by offering support if needed, but also independence, challenge, and learning (Carstens, 1993). Some examples of the translation of such features into design include accessibility (physical and experiential) to the main and secondary areas that lead to different kinds of experiences, the legibility of the layout (what to do and where), the opportunity to make choices, graded difficulties (physical and psychological limits of users), security, respect for anthropometric measures (raised beds that can be easily reached by people on wheelchairs), and microclimatic regulation for use at different times of the day and for most of the year.

Regenerative environment: one which helps to renew or revitalise psychological and physical resources, give new energy, enhance resilience, recover the capacity to fend off distraction and coercion, and renew the cognitive powers of a person (Kaplan, 1983). This concept leads to some specific characteristics of the environment like complexity, richness (biodiversity), sensory stimulation, attractive and more wild-looking destinations, and the opportunity for wandering.

Ecosystem value: one with the enhancement of the ecological functions and benefits that the natural ecosystems provide to support native life forms. “Healthy ecosystems” seem to be more relevant than “nature” for restorative experiences, and it is due to synergic the compatibility between environmental attitudes and healthy ecosystems that triggers restorative processes (Giusti and Samuelsson, 2020). The design should derive from the study of local natural models, focus on nature-based solutions, and aim for multifunctionality and sustainability. The sustainability of a designed garden carries a positive message, perceived at a subliminal level by the user; it communicates the care for the well-being of all living beings (Marcus and Barnes, 1999). An excessively artificial garden that requires continuous maintenance and communicates artificiality and control, on the contrary, is perceived in a negative way.

## Focus Groups for Citizen Engagement

In order to link the general design criteria to the actual experiences of the local population in nature, a co-design approach was developed. The term co-design often includes a wide range of design practises (Sanders and Stappers, 2008). For the purpose of this work, we consider it as a collective task, including the analysis of a challenge in the present context and the attempt to address it through a creative act (e.g., a physical transformation, a new service, or a product) to improve a future context (Zamenopoulos and Alexiou, 2018). In such a perspective, the crucial aspect expressed by this term is in its connective, cooperative, and collaborative nature, which includes both the professionals involved in the design process and involved non-professionals through consultations.

In this general framework, we chose focus groups for implementing the method. A focus group discussion is a technique where a relatively homogeneous group of individuals is purposely selected to provide information about specific topics of interest (Hughes and DuMont, 1993). Focus group discussions are widely used in co-design interventions, but it is not uncommon to find the terminology “focus group discussion” used synonymously with “group interviews” or “workshops” (Hanington and Martin, 2012; Kpamma et al., 2017; Salvia and Morello, 2020). The relationship between the researcher and the participants distinguishes focus groups from other participatory methods. During a focus group discussion, in fact, the researchers adopt the role of “facilitators” or “moderators” rather than the role of “investigators” (Smithson, 2000). Their roles are more peripheral than central (Bloor et al., 2001; Sim, 2002; Smithson, 2007): they are there to moderate and stimulate the group discussion between participants and not between the researchers and the participants.

From this point of view, in the process of the co-design of public spaces, citizens are involved as experts on their own relationships with such spaces (see Table 1). Indeed, they are invited to discuss their own experiences in living or carrying out specific activities in these spaces. The sum of the attitudes, beliefs, and opinions of the participants can provide new critical insights and lead to a greater understanding of the topics of interest (Massey, 2011). The role of the researcher, then, is to facilitate the exchange of personal views among participants, utilising the topics raised to further feed the discussion. Such discussions revolve around personal experiences in or attitudes and beliefs about those spaces, without explicitly asking for design solutions. The kinds of data obtained in this way can be interpreted at two levels: articulated data and attributional data (Massey, 2011). “Articulated data” refer to the explicit answers of participants to issues proposed during the discussion, corresponding to what Braun and Clarke (2006) define as an inductive thematic analysis. Conversely, “attributional data” are theory driven, which means that they are not explored directly but, instead, indirectly, and then categorised. At the second level, consistent with a deductive thematic analysis, psychologist researchers have the responsibility to translate the narrations of participants into consistent and theoretically grounded concepts; designers, on the other hand, are in charge of transforming the results into physical environmental features (Boffi and Rainisio, 2017). The wide availability of data offered by the focus group makes it a preferable methodological choice compared with other co-design techniques, as it favours the integration of theoretical models during the analysis phase. This “biophilic co-design approach” allows us to link general biophilic principles to the specific needs of a local community.

**Table 1:** Actors and roles in the co-design process.

**Actors involved in the process of co-design of public spaces**	**Roles**
Psychologists	Research design, focus group design, and execution (data collection and analysis), informing the landscape design phase
Designers	Research design, informing the focus group design, transforming focus group results into physical environmental choices, design plan, and execution
Focus group's participants	Sharing their personal experience, beliefs, and attitudes

In this study, ART represents a common framework that allows us to build a bridge between psychology and design, thus envisioning the landscape with an experiential approach that relies on the interactions with nature described by participants and integrating the prosthetic aspect of the environment for psychophysical comfort and stimulation. In the light of what has been described, the goals of this contribution are:

- to collect the experiences of different groups of older people in nature to complement the criteria offered by biophilic design with contextual data;
- to interpret such experiences by referring to a specific theoretical model, ART, which is a widely consolidated and employed theory in applied and research contexts;
- to inform the design project through the interpretation of such experiences with the factors of ART;
- to include information consistent with the principles of biophilic urbanism in the design process.

## Method

This article illustrates the co-design process developed for a project that aimed to redesign a portion of a community garden (CG) named “San Faustino,” located in the Ortica district in Milan, as a sustainable restorative natural space for older people. This study area, part of the broader CG, was designed to benefit older people through exposure to and contact with nature. The study area is mainly dedicated to the guests of two nursing homes participating in the research project, even though the CG is open to the public who can also access this area.

The research team adopted a multidisciplinary approach to the co-design process, integrating psychology and landscape design. Local stakeholders were involved in the process, namely, institutional representatives, private nursing homes, and neighbourhood associations. The research activity included the following main steps:

- the development of the research design: the research team developed the materials and methods consistently with the objectives of the study. This included the recruitment of participants, location identification, a site analysis, and a preliminary design;
- the first round of focus groups, which aimed to collect information about needs and desired experiences of users;
- the stimulation of the CG design project by sharing findings from the first-round focus groups with the team;
- the final design project, including related communication materials;
- the second round of focus groups, aimed to gather feedback on the findings from the participants and collect their reactions to the design project.

This section presents the method of the study, including both the design of the CG (the preliminary design and its fine-tuning) and the involvement of the citizens through co-design (materials, participants, and procedures that allowed us to collect data and their analysis criteria). The following section describes the results.

### Site Analysis and Preliminary Design

The first step in the design process, before proceeding with the focus group phase, was the site analysis. This step consisted of research activity that looked at the existing conditions of the project site, along with any potential future conditions. The aim was to identify the values of the place and the critical issues to be mitigated or resolved and to better understand the relationship of the place itself with the neighbourhood context. Therefore, the physical characteristics (e.g., site boundaries, contours, dimensions, site features, and microclimate) and natural features (e.g., typology of vegetation, composition) of the site and the typology of current users were analysed. All the information acquired allowed the designer to understand the existing opportunities and problems in the site. By relating what emerged from the site analysis to the design criteria of a restorative garden, which were previously illustrated, it was possible to develop preliminary design proposals and produce some material for an initial discussion with the participants of the focus groups.

### First Round of Focus Group Discussions

#### First-Round Materials

In order to carry out the focus groups, a semi-structured schedule was built to investigate three specific topics: the image and characteristics of the district (not investigated with the older people hosted in the nursing homes), for example, using sentences to stimulate the discussion, such as “Describe your typical day in the district”; the image and characteristics related to the CG, e.g., “I go there when I want to feel…”; expectations about the study area and the desired experience of living in nature, e.g., “Imagine that, in 1 year from now, the CG is frequently attended by older people: why is it successful?” The image of a place is defined as a mental product that is culturally elaborated, resulting from observation, perception, orientation, and experience; far from being permanent, it is likely to be the result of continuous confrontations, exchanges, and agreements between individuals (Lynch, 1960). Considering our goals, the images of the district and the garden were as important as the experience of the participants in nature.

The semi-structured schedule was integrated by a set of cartographic and photographic materials according to the three topics explored. They included: (topic 1), a map of the Ortica district (A0 size) and postcards reproducing the main landmarks of the district (placed on the table among the participants); (topic 2), four photographs of the study area as it was before the transformation in order to evoke the visual context of the garden according to the principles of experiential simulation in design (Piga, 2011; Piga and Morello, 2015) (A0 size, placed around the participants), a CG map (A1 size), and postcards reproducing the main landmarks of the CG (placed on the table among the participants); (topic 3), a set of postcards representing evocative images of nature or people in nature consistent with the design criteria, for example, an older person smelling a flower, a group of older people chatting on a bench, or even the image of some birds on a tree (placed on the table among the participants).

#### First-Round Participants and Procedure

The first round of focus groups involved carrying out three focus group meetings that were held with potential users of the CG. The participants were recruited through local stakeholders: local administrative institutions, district associations, and nursing homes provided three lists of people aged over 65 who were likely to access the CG due to geographical proximity. Snowball sampling was used for district residents and members of associations. The residents of the nursing homes were recruited with the support of local healthcare personnel, selecting those with cognitive functions to allow a meaningful interaction. The first focus group involved older people living in the Ortica district or nearby (six participants, five women and one man; age range, 65–71; acceptance rate, 43%); a second focus group involved older people actively involved in local associations, even if they were not residing in the district (10 participants, 6 women and 4 men; age range, 65–82; acceptance rate, 100%); a third focus group involved older people hosted in the nursing homes next to the CG (seven participants, five women and two men; age range, 70–84). District residents in the first focus group did not know one another, except for a married couple. The local activists in the second focus group were members, among others, of an association active in the CG. The guests of the nursing homes were acquaintances. Two trained psychologists led all the focus groups to facilitate interactions and took notes during the discussions; the third focus group included two additional facilitators already familiar to the participants. All the participants were Italian native speakers and long-time residents of Milan or, in the third focus group, of close municipalities before moving to the nursing homes. The characteristics of the three groups are consistent with respective target populations, even though district residents were not balanced for gender. No discussed issues or group interactions suggested a related effect. The participants signed written consents, allowing the anonymous analyses of the recorded audio. All the subjects gave their informed consent before participating; the study was conducted in accordance with the Declaration of Helsinki, and the protocol was approved by the Ethics Committee of University of Milan (Project Green Space for Active Living. Older Adults perspectives) on April 19, 2019. The audio recording started after signing the consent, with each focus group meeting lasting ~2 h. The first two focus groups took place in the meeting room of a well-known local cooperative; the third focus group was held in a local nursing home. During the first part of the focus group on topics 1 and 2, the cartographic and photographic materials facilitated the sharing of the personal image of the district and the CG among the participants, as the emerging issues were placed on the maps. In the last part of the discussion on topic 3, the participants were asked to describe their relationship with nature and, through the selection of the postcards with evocative images, to reflect on the kind of experiences they would like to enjoy while interacting with the study area.

#### First-Round Analysis

The data for the analysis included written notes and the comments placed on the maps and postcards by the facilitator or the participants themselves. The audio recording supported the analysis, providing the original context for the data. A thematic analysis (Braun and Clarke, 2006) was performed on the qualitative data collected during the different phases of the focus group, adopting a hybrid approach that combines inductive and deductive analyses (Fereday and Muir-Cochrane, 2006). Data on the first two topics, namely, the image of the district and of the CG, were analysed with an inductive thematic approach; no pre-existing coding frame was applied to identify the main themes introduced by the participants. Such a thematic analysis was integrated with a representation of the themes on the map of the district to emphasise the spatial connexion of each theme with a specific location in the district. The third topic about the desired experiences of the participants in nature was analysed with a deductive thematic approach; the *a priori* codes for classifying the data were represented by the four dimensions of ART (being away, compatibility, extent, and fascination). Each sentence used by the participants to describe such experiences was assigned to a single ART dimension. In some cases, these theory-driven themes were further divided into data-driven sub-themes to track relevant aspects of the results, thus informing the design project (e.g., for the compatibility and fascination dimensions). The same approach was carried out by the psychologists for all the focus groups and subsequently interdisciplinary validated.

### Fine Tuning of the CG Design Project

The results of the first-round focus groups were the basis for a multidisciplinary exchange, stimulating the revision of the initial design in consideration of the experiences collected. The four factors of ART were the basis for the discussion and the implementation of the final version of the design.

### Second Round of Focus Groups

#### Second-Round Materials

For the second round of focus groups, a new semi-structured schedule was designed: the first part aimed at presenting the results of the previous phase and the design proposal; the second part aimed at investigating the reactions to the designed study area, for example, using sentences stimulating the discussion such as “What elements of the design projects impressed you the most? What does it remind you of?”; the third part aimed at exploring the image of the renovated CG, e.g., “Imagine you are describing the CG to a friend….”

As in the previous round, in addition to the semi-structured schedule, the procedure included additional cartographic and photographic materials for facilitation: four pictures of the study area as it was before the transformation (A0 size, placed around the participants); four pictures of the study area according to the design project to evoke the future condition (A0 size, placed around the participants); a map of the study area renovated according to the design project (A1 size); a set of postcards reproducing the main elements of the design project; a CG map, including the study area renovated according to the design project (A1 size); a set of postcards reproducing the main landmarks of the CG (existing and renovated).

#### Second-Round Participants and Procedure

The second round of focus groups involved the realisation of three more focus group meetings, one for each target group of potential users engaged during the first round. Contacts collected for the first round, including the former participants, were invited again. The first focus group with older people living in the Ortica district or nearby included three of the former six participants, and no new member joined the group (three participants, two women and one man; age range, 65–70; acceptance rate, 23%). The second focus group with older people involved in local associations included all the former participants (10 participants, 6 women and 4 men; age range, 65–82; acceptance rate, 100%). The third focus group with the guests of the nursing homes included all the former participants and five new members (12 participants, 9 women and 3 men; age range, 70–84). The characteristics of the groups and their influence on the discussed issues were comparable with those observed during the first round. The same facilitators were involved, including the landscape designer for the first phase of the focus groups.

During the first phase of the meetings, the participants were presented with the results of the first round of investigation to reinforce their active roles in the process. During this phase, the room in which the focus group took place was set up to evoke the current visual context of the project: four photographs of the study area before the transformation were placed around the table of the focus groups. At the end of the presentation, the four pictures around the participants were substituted by four pictures of the study area according to the revised design in order to evoke the future condition. At the same time, the map of the study area according to the design project was placed on the table. The landscape designer introduced the biophilic design principles and showed the features of the design solution. After answering a few questions from the participants, the designer left the room, and the second phase started. The participants were asked to indicate which elements impressed them the most and why, providing explanations for what they considered specific strengths or weaknesses according to their experiences in nature. In order to support this part of the process, postcards illustrating the main elements of the project were used. The third and last parts of the focus group meetings were dedicated to exploring the prefigured image of the CG, considering both current and future elements. To support this phase, a map of the CG was proposed, including the study area according to the design project.

#### Second-Round Analysis

The data construction and analyses for the second round followed the same criteria as those previously described. An inductive thematic analysis was performed on the experiences in nature elicited by the design project, collected during the second phase. A deductive thematic analysis was carried out on the image of the renovated CG explored during the third phase.

## Results

This section presents the results of the first-round focus groups, their impact on the design proposal, and the results of the second-round focus groups. As the main interest of this study is the integration of data on the experiences of older people in nature into the entire design process, the emphasis is on the most relevant results obtained in each phase and their use in the subsequent phases. For a more detailed analysis of the first-round focus groups, see Fumagalli et al. (2020). From the first-round focus groups, we present:

- a description of the image of the Ortica district, where the CG is located, and the green areas identified as landmarks by the participants;
- a description of the image of the community garden where our study area is located;
- expectations about the study area and desired experiences of potential users in nature with the study area.

From the second-round focus groups, we present:

- the coherence between the desired experiences that emerged from the first-round focus groups and the study area according to the design project;
- the CG image in the light of the introduction of the study area according to the revised design project.

### First Round of Focus Groups

#### The Image of the Ortica District and Its Green Areas

**Figure 1:**
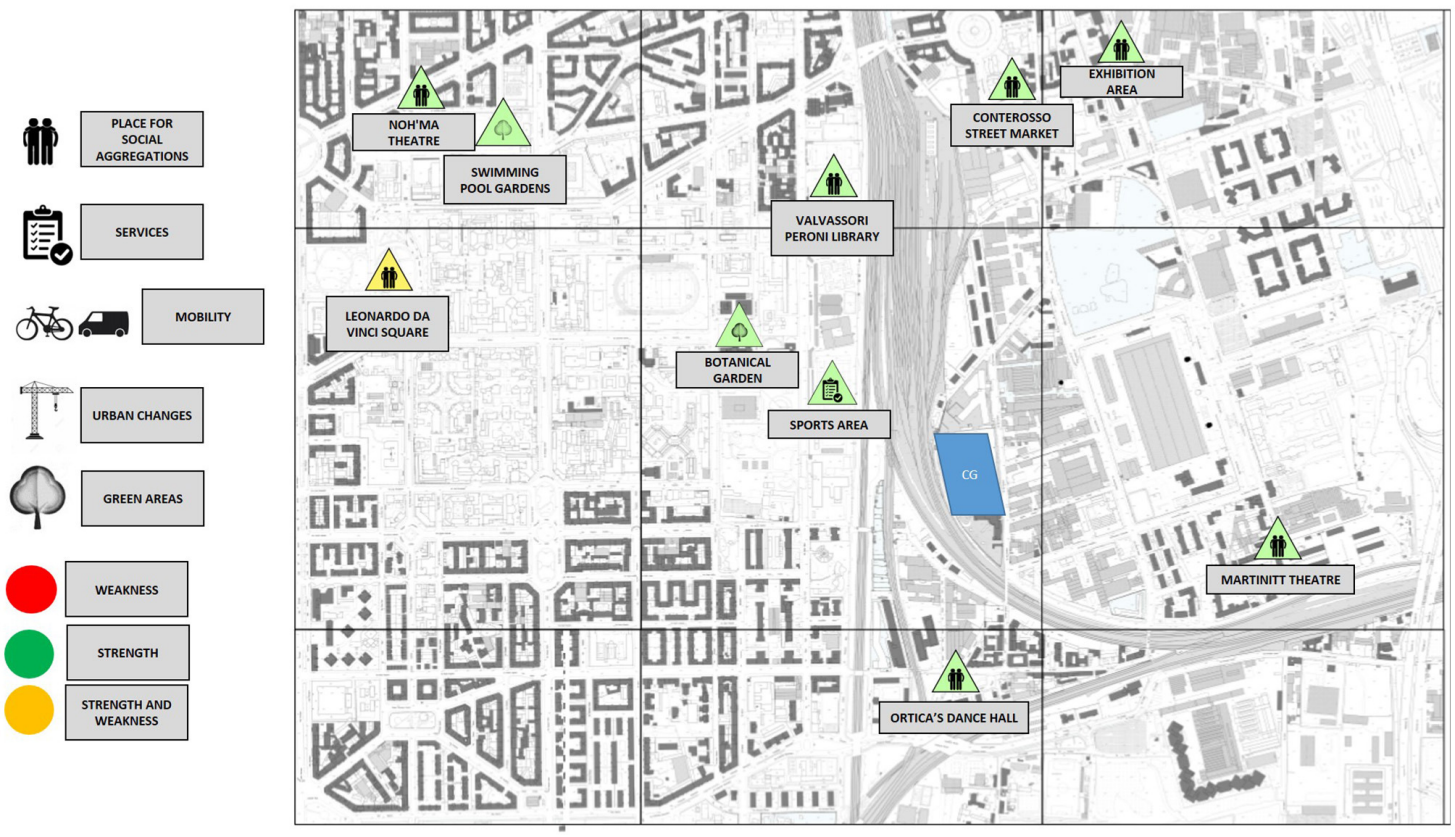
A map resulting from the older people living in the Ortica district or nearby. The community garden (CG) position is marked in blue.

**Figure 2:**
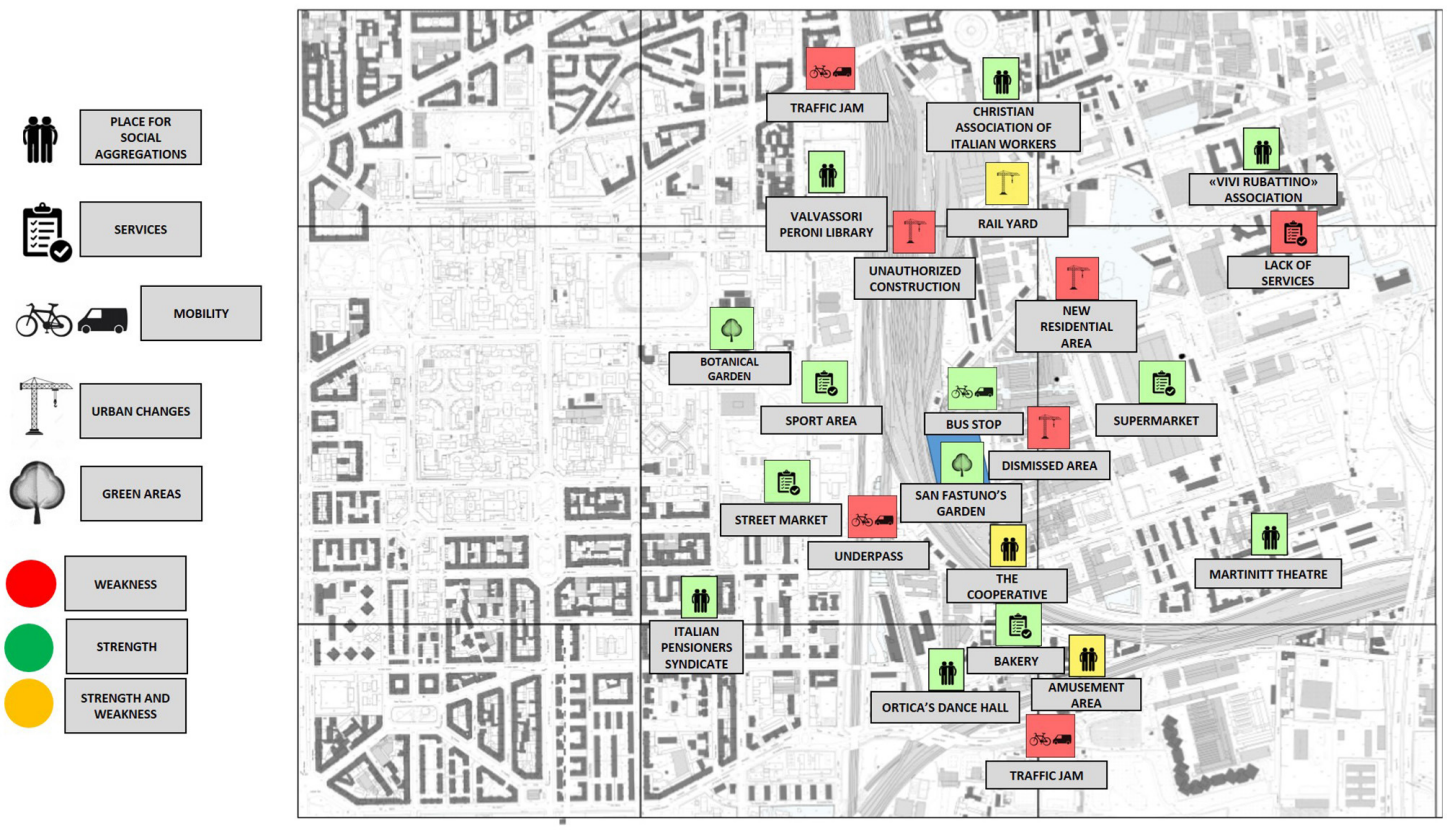
A map resulting from the older people actively involved in local associations. The CG position is marked in blue.

The image of the Ortica district that emerged from the personal experiences of the participants allowed us to identify the distinctive features of the place, both physical and symbolic. The results are summarised in Figures 1, 2. Each place mentioned during the discussion was included in a bottom-up taxonomy composed of places for social aggregation, services, places relevant to mobility issues, places of ongoing urban transformation, and green areas. In addition, each place was categorised as a strength, a weakness or a 2-fold issue, including both strengths and weaknesses.

The maps resulting from such a classification highlighted two very different perspectives. Older people actively involved in local associations, although not all living in the Ortica district or nearby, showed greater knowledge of the strengths and weaknesses of the area. However, there was a general lack of awareness of the available resources and of weaknesses of the district from the older people living in the Ortica district or nearby. This general level of knowledge was associated with the frequency of reported access to local green areas. In fact, even if green areas are considered as a general positive aspect of a district by both groups, older people living in the Ortica district or nearby did not report spending much time there. Two green areas were mentioned by the older people living in the Ortica district or nearby, namely the botanical garden, which is identified as a place mostly suitable for children, and the greenery surrounding the local swimming pool, which is associated with the café service without mentioning the natural experience itself (“*Sometimes, just to have a quick walk around, I go to the pool garden, where there is a café”*). Both of these places were frequented sporadically. The older people actively involved in local associations also described a district with few green areas, but, unlike the older people living in the Ortica district or nearby, they had daily routines where the relationship with green elements was more present. In addition to the botanical garden, the older people actively involved in local associations also cited the CG, where they acted as volunteers [“*We wanted to work in a green and beautiful place that made us feel good. I studied agriculture and it seemed nice to put my knowledge into practise at the service of citizens* (...) *We also have very nice exchanges, nice discussions”*].

This evidence has an important impact on the co-design process of the study area. For those citizens living in the area but not actively involved in local associations, the district where the CG is located is, overall, hardly considered to be one of the available resources that satisfy personal needs, even if no specific critical aspects are mentioned. However, they cited a few local places for social aggregation, a few green areas, and almost no services (“*My typical day? I walk around with no destination”*). Although the district is recognised as undergoing a requalification process, older people living in the Ortica district or nearby did not mention the transformation as a chance to meet their own personal needs. In such a perceived void that characterises the area, the new regenerative garden included in the CG becomes less salient. Indeed, for older people living in the Ortica district or nearby, the CG is in an almost empty space, without meaningful places that function as attractors or reference points to reinforce its appeal. The district map resulting from the perception of older people who are actively involved in local associations offers, instead, a complex and multifaceted representation. They show a deeper knowledge of local urban spaces and their transformations. Such an aspect is visible on the map in quantitative terms (a higher number of salient elements placed in the area), in qualitative terms (a wider spectrum of categories of the taxonomy; a more nuanced list of strengths, weaknesses, and 2-fold issues), and in spatial terms (a more homogenous distribution of the elements throughout the area with no empty spaces). For the older people actively involved in local associations, the space surrounding the CG is richly populated by meaningful places, which are not always positive but are a vivid source of debate that suggests the relevance of the area in their perception.

#### The Image of the Community Garden

The first round of focus groups also allowed us to characterise the CG where the study area is located. Consistent with the perceived void in the district, the CG was barely known by older people living in the Ortica district or nearby. However, the older people actively involved in local associations and the guests of the nursing homes had good knowledge of the CG and could highlight which elements of flora, fauna, human artefacts, and other elements characterised their imaginations about it. Regarding the plants, most of the participants mainly mentioned trees (e.g., cherry and elm) rather than other greenery. The trees were seen as key elements characterising the site in contrast with the urban context (“it is the background that gives you the idea of not being in the city”). Various animals were cited, mainly in positive terms (e.g., pheasants and bees), even though mice were seen as potentially damaging for the area. The discussion about human artefacts often highlighted a 2-fold perception, as they were perceived as positive elements but could not adequately manage to fulfil the potential of the garden (e.g., the vegetable gardens should be kept in a smaller dedicated area, the artificial pond should be refurbished, and the hives were not working properly); the arbour was the only element perceived as completely positive. Finally, some general concerns were expressed about mobility within the CG. For further information about the results that emerged on this topic, see Fumagalli et al. (2020).

#### Expectations About the Study Area and the Desired Experience of Living in Nature

The results regarding the prefigured experience that the participants associated with the study area have been discussed in depth in Fumagalli et al. (2020). A summary of the main findings is presented here and in the first row of Tables 2–5, separately for each ART factor, in order to provide a more complete overview of the results of the co-design process.

**Table 2:** Comparison of the needs and reactions of the participants to the design proposal [attention restoration theory (ART), compatibility].

**ART dimension: compatibility**	
Needs emerged in first-round focus groups	1. To have an aesthetically pleasant place for intra-generational and inter-generational social exchanges (“*If there is beauty, the possibility of interpersonal exchanges is interesting”)* 2. To have a place for knowledge in itself (“*I like to look at plants [...]Find out whichplants are there”*) 3. To have a place to implement manual skills (“*An area where you can do your vegetable garden [...]Hand activities for plant care*.”)
Design solutions	1. Although mainly dedicated to the older people, the whole garden is attractive for the different generations to facilitate inter-generational social exchanges. Every design element, linked to active or passive activities was made available to users with high levels of physical limitations, also to guarantee the older people's autonomy and ease of orientation (visible paths, landmarks, clear visibility of the whole garden). 2. An illustrative panel, at the beginning of the main path, illustrates the layout of the area with the main attractions, the strategic design elements and specified their functions. Main trees are identified with a common name tag for an easy recognition 3. The manual activities mainly concerntaking care of the plants, watering them, weeding, but without a specific commitment. This freeuse of the space allows visitors to contribute to its maintenance and care(that are formally entrusted to a specialised company, technical partner of the project). The garden does not propose specific activities, such as horticultural ones, as it is designed as a restorative garden where there are no prescribed therapeutic or occupational activities
Designed elements highlighted in second-round focus groups	1. The pergola (Supplementary Figure 1) is recognised as the design element most suitable to satisfy socialisation needs. The participants show some doubts about its comfort. In this regard, some participants propose to implement the pergola through some form of covering, to allow people to use it even during less favourable weather conditions, such as wind or rain. Another potential weakness in this area is an adequate shading *(“the trees that you will plant are already big, right?”*). Participants seem to appreciate also the design philosophy introduced to them, about designing a place open to everyone (“*This is the spirit… design for all*”). They also highlight as a strength the design solutions intended to favour an inter-generational use of the space (Supplementary Figure 2) 2. To respond to this need, participants propose to add information plaques about the variety of plants in the study area *(“will there be a card for each plant? -...someone entering the garden wants to find out new information”*) 3. The designed area is prefigured to respond to the need for doing manual work on the greenery *(“I imagine myself going there to do manual activities, such as using the mower”*). This expectation is not specifically linked to any of the design elements presented

**Table 3:** Comparison of the needs and reactions of the participants to the design proposal (ART, fascination).

**ART dimension: fascination**	
Needs emerged in first-round focus groups	1. Being in contact with flowers (“*It would be the great to have pathways through the flowers”*) 2. Being in contact with fruits (“*it would be nice to also have spontaneous fruit trees”*) 3. Being in contact with animals [“*if older people, who have a dog or a cat, found small animals in the garden (...) for those who have not lived in the city but have lived in the countryside it is like returning to their roots”*] 4. Being in contact with aquatic elements (“*it would be nice to have a small body of water”*)
Design solutions	1. The design included large areas of wildflowers along the main path, a sensory garden with numerous brightly coloured blooms, two raised flower beds with edible and cut flowers and several hedges of ornamental shrubs that seasonally and gradually produce abundant blooms 2. The project included an orchard, along the main path, a row of mulberry trees and a naturalistic hedge with native shrubs that produce wild fruits, for birds and small mammals, but also edible for people 3. Many design elements serve as habitats and food reserves for mammals, pollinators, and beneficial insects. The project included a bird garden with bird feeders and a hedge of mixed native berry-producing shrubs, beneficial to birds 4. The project included a small, clearly visible, and accessible coloured water fountain. For technical and maintenance reasons it was not possible to insert a pond or a water basin
Designed elementshighlighted in second-round focus groups	1. The design project of the study area includes an area dedicated to flowers (Supplementary Figure 3). This element is considered an important and positive element responding to the need of the potential users (“*It is beautiful… my soul could be blessed with all that beauty, all those perfumes*.”). In this regard, participants propose to increase the close contact with flowers, offering the possibility of being surrounded by them (“*I would like to lie down in the middle of this meadow in bloom”)* 2. The orchard area (Supplementary Figure 4) is very appreciated *(“It's very important for me to have fruit trees… it represents even more clearly the fruitful interaction between nature and human being”; “stay in an orchard means to be in a living space)”*. Some participants underline concerns about the salubrity of the ground (“*it was an industrial area… would it be safe to eat fruits grown there?”)* 3. The presence of elements such as the bird garden (Supplementary Figure 5) and the Benje's hedge (Supplementary Figure 6), finalised to attract and protect the local animals, has been underlined as a strength. Yet, there are concerns about these same elements. Respectively, participants point out how the bird feeders may also attract pigeons (“*I like this project element, as long as the pigeons do not come*”) and they seem worried about the time needed for the Benje's hedge to respond to its function *(“How long do we have to wait… years?*”). In general, the presence of mosquitoes also emerges as a potential weakness, so the introduction of bat houses is proposed as a mitigation action 4. Even if the presence in the project of a drinking fountain (Supplementary Figure 7) is recognised as positive (“*The pink drinking water seems cool!!*”), the lack of additional water elements is seen as a disadvantage (“*For example, in the botanical garden there is a pool of water; water is important for children and people.”; “there was a pond here. are there frogs?”)*

**Table 4:** Comparison of the needs and reactions of the participants to the design proposal (ART, being away).

**ART dimension: being away**	
Needs emerged in first-round focus groups	Being away from everyday life [“*A place for reflecting, stopping and contemplating (...) Even being alone just for an hour would be nice to me”*]
Design solutions	The first design action was to identify the major disturbing elements in the surrounding urban contextand reduce their impact. Vegetal structures have been introduced to mitigate or screen the neighbouring buildings, enhancing more pleasant views. The second step was to design an interesting and pleasant setting (lush and various vegetation), inviting to interaction (sensory garden, bird garden, natural playground etc,) and to exploration (more wild areas), accessible to all kind of users (a main path in permeable paving and an “explorative” secondary one on mown grass), where it is possible to do different activities (multifunctional)
Designed elementshighlighted in second-round focus groups	The designed area is appreciated for its ability to respond to this type of need (“*it's like an oasis, you feel far away from the city; I would even go there alone, for contemplation”*). Coherently all the participants mention as a positive aspect the introduction of a mitigation area designed to hide the presence of the adjacent nursing home *(“It is essential to cover that building. It is a punch in the eye.”*)

**Table 5:** Comparison of the needs and reactions of the participants to the design proposal (ART, extent).

**ART dimension: extent**	
Needs emerged in first-round focus groups	No needs emerged
Design solutions	The width of the area and the presence of adult trees within the CG are, at the design level, an important starting point to enhance the “extent”factor. Every area is physically and visually connected; two different paths allow to explore the whole garden easily and safely; the layout of the area is legible and the message of what can be done and where is clear. The vegetation and architectural elements that build the restorative setting contribute to the functioning of the place and to the feeling of being immersed in coherent and unique environment
Designed elementshighlighted in second-round focus groups	During the second-round this dimension of ART is defined more precisely. The designed study area is perceived to have internal coherence and to be suitable for orientation between the different designed zone. In particular, the presence of a double path is appreciated by the prefigured users. Yet, some mobility concerns are expressed about the accessibility of the main path through the garden for older people using walkers or wheelchairs

Compatibility emerges as the ART dimension most prefigured across the groups, although the analysis underlines the differences among them. Compatibility, which is connected to the possibility of using the area as a place for interpersonal exchanges, is particularly important in the imaginations of the older people living in the Ortica district or nearby [“*This could be an exchange area. It's refreshing to be in contact with other generations. It's a joy to see the kids* (...) *This dimension is more important to me than just relaxing”*]. Compatibility appears to be less relevant for the older people actively involved in local associations and for the guests of the nursing homes. In this regard, we must recall that, as presented in the previous paragraphs, the older people living in the Ortica district or nearby find it difficult to identify significant places within their social context that can meet their socialisation needs [“*An outdoor-listening group could be instituted* (...) *Many older people just need to talk*”]. On the contrary, the map of the older people actively involved in local associations identifies various places of socialisation in their daily routines in addition to the CG. Finally, concerning the guests of the nursing homes, a wide range of activities is planned daily for indoor spaces shared by the guests [“*Here, there are already groups to be in the company of others, while I would like to sit there and see the leaves move* (...) *reconnect with nature”*].

The ART dimension of fascination is the second most relevant experiential dimension associated with the study area, according to the desires and prefigurations of the participants. A multisensory experience related to contact with natural elements is highly desirable for the targets [“*The greenery does not involve only the sight but even other senses* (...) *It attenuates noises*(…) *Especially for me, since I am very anxious and nervous, it is very important”*]: being in touch with the colours and scents of flowers and fruits, with the vitality of plants, animals, and aquatic elements, was widely identified by the participants as the kind of experience in nature most capable of giving them pleasure, joy, and peace.

The third most important dimension of ART is being away. The opportunity to escape from everyday life through immersion in a green space is important for all the targets interviewed, although it seems to be a particularly relevant experience for the guests of the nursing homes. In fact, the older people living in nursing homes seem particularly sensitive to the desire for escape as a reaction to the perceived contraction of their space-time freedom and physical skills [“*We would use it just to break out a little bit* (...) *it would be the only opportunity to go out for a little bit”*; “*I would like to enjoy nature alone by myself”*].

Finally, the extent, linked to the need for cohesion between the elements that compose a natural landscape, seemed to be the least important dimension. It is worth noting that the study area is small, that could make the need for feeling safe while exploring less relevant.

### Design Proposal

The needs that emerged from the first round of the focus groups were compared with the preliminary project. On the basis of the collected information, the design solutions were realigned or adapted, accommodating, as far as possible, the necessities of users. The experiential ambitions in the garden emerged from the focus groups are summarised in two trends in terms of design:

(1) a multifunctional garden compatible with the needs and attitudes of the identified targets, consistent with the compatibility factor of ART: a place (a) of aggregation and relational exchanges, b) for manual activities, and (c) for and where to find cultural initiatives;

(2) a garden that can create contact with nature, consistent with the being away, extent, and fascination factors of ART: a place (d) of fascination and mystery, (e) where other kinds of experiences from everyday life can be lived, and (f) for concentration and solitude.

The contextual information about the image of the district allowed us to weigh the perception of the CG by different groups, supporting the creation of a synthesis to inform the design proposal. One of the most relevant aspects in this regard was the factor of compatibility. The participants living in the district assigned it great importance, imagining many activities to carry out in the CG. Yet, many of them could be consistently satisfied by other facilities, which already exist in the district but of whose existence they do not know. This prominence given to compatibility is combined with the lower relevance assigned to being away, which is, instead, the most important aspect for the guests of the nursing homes who have few alternatives for experiencing it. The combined interpretation of the data on the district and the CG led the research team to reduce the weight of compatibility, as many of the needs expressed by residents could be addressed by other types of interventions in the district.

The design process, firstly, developed a concept plan that defined the functional areas (what to do and where) and their connexions to ensure the efficient use of the space, and secondly, a master plan that specified works in their entirety. A summary of the main design choices is presented in the second row of Tables 2–5, separately for each ART factor and consistently with the contents that emerged from the first round of the focus groups. The project was represented through tables and realistic renderings that were as similar as possible to the actual desired result for clear and legible communication.

### Second Round of the Focus Groups

#### Coherence Between the Desired Experiences That Emerged in the First-Round Focus Groups and the Designed Study Area Evaluation

The results of the second-round focus groups concern the reactions to the design proposal for the study area. The contents are categorised according to the ART dimensions, and a summary of the main findings is presented in the third row of Tables 2–5 separately for each ART factor and consistently with the results of the first round and the related design elements (these are included in the Supplementary Materials). It includes references to the postcards of the main design elements of the project, which was proposed as a tool for discussion. The postcards are reproduced in the Supplementary Materials. In general, the needs of the first round of the focus groups are met according to the inductive thematic analysis based on the ART factors. Overall, the factor of compatibility appears satisfied in its diverse components (the pergola for social relations; the labels describing the plants for knowledge needs; the free access to taking care of the plants for the desire for manual activities). Some weaknesses are mentioned, specifically regarding the comfort experienced under the pergola for temperature (shade to protect from the sun) and bad weather (wind, rain). The elements classified under the fascination factor are all positively evaluated: the contact with flowers, fruits, animals, and water is recognised as valuable. The most debated negative aspects regard health issues (possible pollution of the soil connected to the fruit trees), the presence of undesired animals (pigeons and mosquitos), and the lack of more water elements (the drinking fountain is considered positive but not sufficient). Being away is appreciated by the participants, and no downsides are mentioned. The factor of extent did not emerge during the first round, yet it was included in the design proposal according to the design criteria; interestingly, the reactions to the design project elicited some comments about the increased coherence of the area.

#### The Image of the Community Garden in the Light of the Introduction of the Study Area According to the Design Project

A further goal of the second-round focus groups was to understand if and how the introduction of the designed study area inside the CG changed its image. The positive elements that prevail in defining the identity of the CG, in general, are the possibility of living contemplative experiences of being away from everyday life and of finding real contact with natural elements (“*For me, the CG would be to breathe and admire the flowering, the autumn with its colours, winter, and every season;” “I also would go there to get out from the community: even if socialisation is important, solitude for me means a sense of liberation;” “what attracts the most is that it's like seeing a piece of the countryside in an urban area”*). On the other hand, the participants underlined the CG location as a negative element; it seems to be far away from frequented paths of their daily lives.

In addition to these general points, the adherence between the image of the CG and the designed study area has been the object of further examination, mentioning what appears to be a general reduction of extent. In this regard, the study area is perceived to be so well-designed that it could make the other areas look a bit worse *(“now, you have to fix the rest!;” “it's a more fancy area, the rest is very wild...”*). Another difference that made the new identity of the CG less homogeneous than before is that the study area appeared to some to be less inviting to an active interaction with nature (“*Our experience in the new area will be much less interactive; we will be afraid to take a flower. In our area, we have an interaction, because we feel it belongs to us*,” said by a participant involved in local associations).

## Discussion and Conclusion

The current study illustrates a case study for the application of a co-design method, which employs biophilia principles, and in which the impact on the participants is assessed using the four dimensions of ART. The method shows that the biophilic design project presented to the citizens elicited an experience of the natural environment that is consistent with the needs collected in the first phase. Thanks to the semi-structured schedule and the deductive thematic analysis, it is possible to describe how the design project answers mainly to the needs of compatibility and fascination, whereas being away and extent are less relevant for citizens critically observing the design proposal, which is consistent with their expectations of experiences in nature. A key feature of the method is the possibility of linking such factors to specific design elements included in the design project by assigning physical elements to the attributional categories used for the analysis; hence, the components of the designed study area in the CG are categorised through the four dimensions of ART, relying on perceptions of the participants. The integration of articulated data allowed us to add observations on specific functional aspects that, even if less relevant in terms of restoration, can be considered for the comfort of prospective users. Another distinctive characteristic of the method is that it places the specific study area into a wider context, offering a representation of the image of the neighbourhood and the CG. This offers some further elements for the development of the design project, which can either affect its physical features in the design phase or inform the guidelines for the effective usage and maintenance of the site in the management phase (Niemelä, 1999; Cadenasso and Pickett, 2008; Philips, 2013). The method is conceived as a practical tool to apply with a multidisciplinary team when designing green areas.

Our results have some limitations that require reflection when considering the applicability of the method. In particular, as the efficacy of the method is evaluated on a single case study, some aspects are worth mentioning. In the first place, the landscape designer involved in the project is part of the research team and has a background in biophilic design. Such conditions are both desirable in our conception of a multidisciplinary approach to co-designs, but we cannot consider them as the standard circumstances observed in the professional field. As a consequence, the capability of the average practitioner to design a garden following biophilia principles can vary, and this can significantly alter the design outcome that is then tested during the second round of the focus groups. In addition, the interpretation of textual feedback categorised through the four dimensions of ART and their usage to fine-tune the design project is part of a broader professional skill; such “ambiance empathy” allows the effective merging of environmental features and human experiential factors and is also significantly different depending on the background of the practitioners (Piga, 2021). Hence, it is also desirable to apply the method with a broader and more heterogeneous sample of landscape designers to appreciate its actual impact. In the second place, the current design project concerns a community garden with a specific focus on older users, including the guests of nursing homes. The issue of active ageing is a crucial aspect informing the development of the design proposal. With this perspective, two main classes of users are targeted; older people, both from the general population and from the nursing homes and who are the primary users, and their related family members of different generations (siblings, children, and grandchildren) and health assistants, who are secondary users. Notwithstanding the heterogeneous population, the central issue of active ageing contributes to the narrowing of the design goals. This aspect is also emphasised by the relatively small dimensions of the area. It is necessary to test the method in more varied contexts and in bigger areas, where the needs can differ to a larger extent and the identification of a main design goal is more challenging. In this case, it would be possible to understand how the method helps in such a process.

Despite such limitations, we argue that the method offers some useful elements for a multidisciplinary approach to co-design on three different levels. The first level regards the importance of human-centred design (Bazzano et al., 2017), which places the experience of an individual at the centre of the design practise and emphasises the importance of empirical data. This helps professionals keep their focus on the needs of people, relying not on general assumptions but on contextual data from actual users and integrating environmental aspects with psychosocial factors. As discussed, this is a key aspect that can reinforce the interaction between the social sciences and design sciences by creating a common ground. It is crucial to remember that, according to the methodology proposed, involving the citizens does not mean assigning them the role of analysing the current context or designing prospective solutions. They are, instead, invited to share personal behaviours, attitudes, and beliefs that reflect the personal meaning they assign to the stimuli offered for discussion.

The second level is related to the choice of the specific theoretical model to describe the experience of an individual that, in this case, is represented by ART. It proved to be a fruitful grid for interpreting the experiences of participants and provided insights and feedback for the designer, also because such a model is effectively rooted in both disciplines (see Fumagalli et al., 2020 for more details of this analysis). All the contents included in the deductive thematic analysis fit with the ART model, even though the theoretical category of extent did not include any content during the first round. This notwithstanding, the main advantage of such approach is in offering a categorisation of both subjective experiences and design elements linked to a well-established theoretical model, which facilitated comparisons across different project sites and design teams. Relying on pre-defined categories reduces the influence of idiosyncratic interpretations. Hence, having a proper theoretical model as a reference allows teams to make specific hypotheses and test them with qualitative and quantitative data using already established methods, which is a remarkable advantage compared with using an inductive thematic analysis. Other valuable options consistent with the biophilic approach are available in psychological literature. The most investigated construct linking psychological and design research is restoration, which, in the first place, guided our choice for the theoretical framework to structure the attributional analysis. A useful alternative in such a perspective is, hence, the stress recovery theory (SRT) (Ulrich et al., 1991). However, another psychological construct less examined by biophilic design literature that can be usefully included in such methodology is well-being. Interestingly, whereas the construct of restoration is strongly integrated into design research and practise, the concept of well-being is often mentioned as a relevant goal for design and active ageing but seldom by referring to a specific construct (e.g., see Bazzano et al., 2017; Istat, 2020). As many disciplines define such notions in different ways, referring to both objective indicators and subjective measures, the diffusion of specific psychological constructs is largely reduced in comparison to other fields of research. Often, “well-being” is most easily understood in terms of comfort when referring to urban planning and design. In psychological terms, it is a rather well-established construct composed of multiple aspects, which are traditionally separated at least in two dimensions regarding hedonic pleasure and eudaimonic self-realisation (Ryan and Deci, 2001). Like restoration, well-being can be conceived as an individual measure that can be assessed and compared with other indicators, both objective and subjective. Indeed, when we think of the role of the social sciences in informing decisions affecting the transformation of the physical environment, having data on psychological assessment offers a clear advantage; “city planners may face choices between economic growth and increased air pollution. Certain political ideologies call for an emphasis on economics, whereas other ideologies call for an emphasis on other factors such as equality or environmental conservation. How can a policy maker weigh alternatives in a systematic way and move beyond a total reliance on intuition and ideology? Broad measures of well-being can provide a valuable source of information because they can reveal how well-being of people is affected by the various policies” (Diener et al., 2009, p. 54–55). However, it is important not to misinterpret such an approach as the idea of policies simply maximising well-being (Stutzer, 2020) or any other psychological construct, including restoration. Such a technocratic approach would be detrimental for an effective participatory approach, consequently diminishing the importance of developmental factors favouring well-being (Prilleltensky et al., 2001; Boffi et al., 2016) and neglecting the way institutions and inclusive procedures increase the value of procedural utility, which is a source of subjective well-being (Stutzer and Frey, 2006). Consistently, with this approach, an eudaimonic perspective on well-being would allow assessments, informing the design of places that are not only restorative from a cognitive point of view but also offering the possibility to reach meaningful objectives for the growth and realisation of an individual (Rainisio et al., 2015; Boffi and Rainisio, 2017).

The third level concerns the instrument adopted for the process. Existing measurement tools for ART are mainly quantitative (e.g., Hartig et al., 1997; Pasini and Berto, 2007; Pasini et al., 2014), which implies the administration of scales to the participants involved in the process. Such an approach has both positive aspects (e.g., brief administration, if not associated with other scales; quantitative data; a more easily replicable data collection) and downsides (e.g., the need for a higher number of participants; a pre-defined set of items that may not best fit the specific context; less space for emerging data), which must be accurately taken into account when designing an engagement process that is psychologically sustainable for citizens (Boffi et al., 2015). Our attempt was, instead, to develop a qualitative tool that allowed us to collect two types of data at the same time. “Articulated data” concern the explicit aspects investigated during the focus group discussion (e.g., landmarks and distinctive features of the Ortica district, which emerged during the focus group), which are self-evident in their content but must be organised in fruitful, bottom-up categories during the analysis depending on the exploratory goals. “Attributional data” are, instead, strictly linked to a specific theoretical model, and their structure is not manifested to the participants but is applied by the researcher (e.g., the four ART categories presented about the desired experiences in nature). The combination of these two categories offers a wide array of content that can simultaneously inform the designers from different perspectives.

In our view, the applied methodology is not meant to be an automatic process for directly obtaining final design solutions. It is, instead, conceived as a transcultural and trans-environmental approach to serve as a logical framework, the specific contents of which are meant to be adapted according to the context, i.e., sociocultural variables, environmental aspects, and design goals. Further developments of the method should also include the following phase of a post-occupancy evaluation to assess the actual impact of the natural solutions on experiences of peoples. Maintaining a consistent theoretical approach and instrument choice would allow a complete monitoring process during all the design phases.

## Data Availability Statement

The datasets presented in this article are not readily available because data include an audio recording of the focus groups that would allow the identification of participants. Requests to access the datasets should be directed to Marco Boffi, marco.boffi@unimi.it.

## Ethics Statement

The studies involving human participants were reviewed and approved by Ethics Committee of University of Milan. The patients/participants provided their written informed consent to participate in this study.

## Author Contributions

NF, GS, PI, and MB: conceptualisation and methodology. MB and LP: formal analysis and data curation. EF, MB, and LP: investigation. MB, EF, and LP: writing—original draft preparation. NF and MB: writing—review and editing. EF: visualisation. PI and GS: supervision. funding NF: acquisition. All authors contributed to the article and approved the submitted version.

## Funding

This work was supported by Fondazione Cariplo (Ageing and Social Research: People, Places, and Relation, 2018).

## Conflict of Interest

The authors declare that the research was conducted in the absence of any commercial or financial relationships that could be construed as a potential conflict of interest.

## Publisher's Note

All claims expressed in this article are solely those of the authors and do not necessarily represent those of their affiliated organizations, or those of the publisher, the editors and the reviewers. Any product that may be evaluated in this article, or claim that may be made by its manufacturer, is not guaranteed or endorsed by the publisher.
