# Supplementary material for: Nature Experiences of Older People for Active Ageing: An Interdisciplinary Approach to the Co-Design of Community Gardens
Source: Front Psychol. 2021 Sep 27;12:702525. doi: 10.3389/fpsyg.2021.702525 (PMC8503679; doi:10.3389/fpsyg.2021.702525)
Supplement: Supplementary file 1 [file Table_1.docx]

Supplementary Material


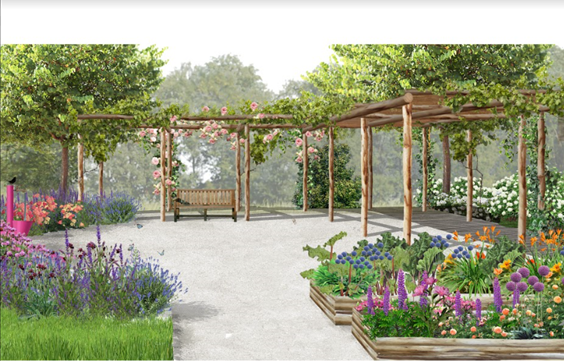


Figure 1 The Pergola


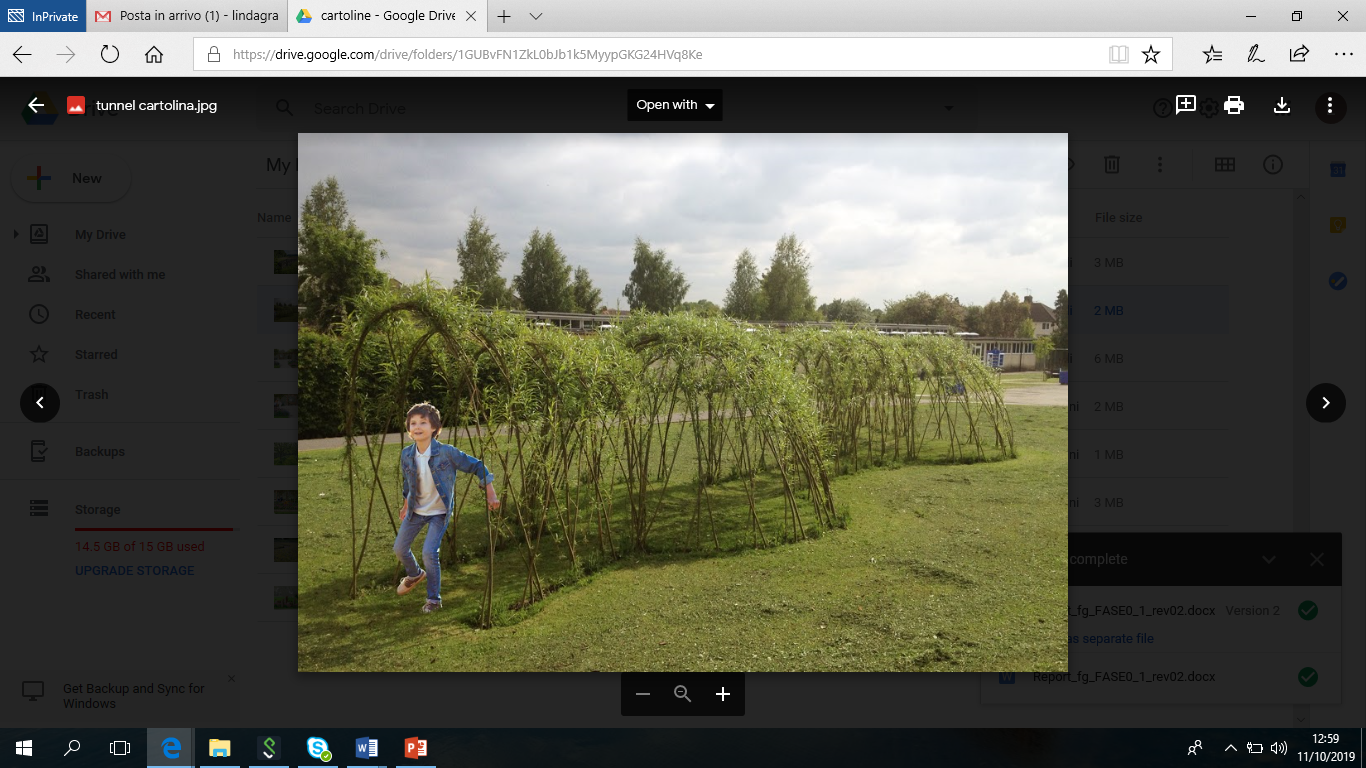


Figure 2 Natural playground


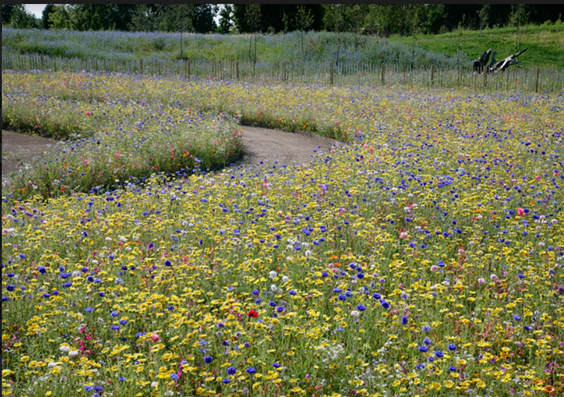


Figure 3 Flowery lawn


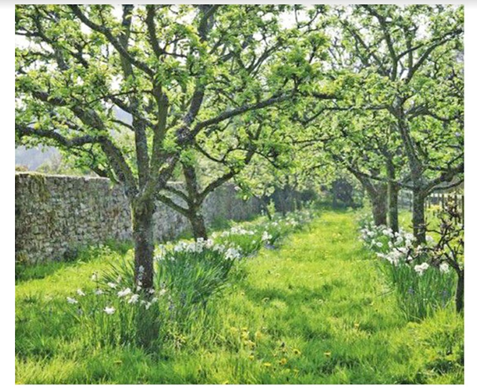


Figure 4 The orchard area


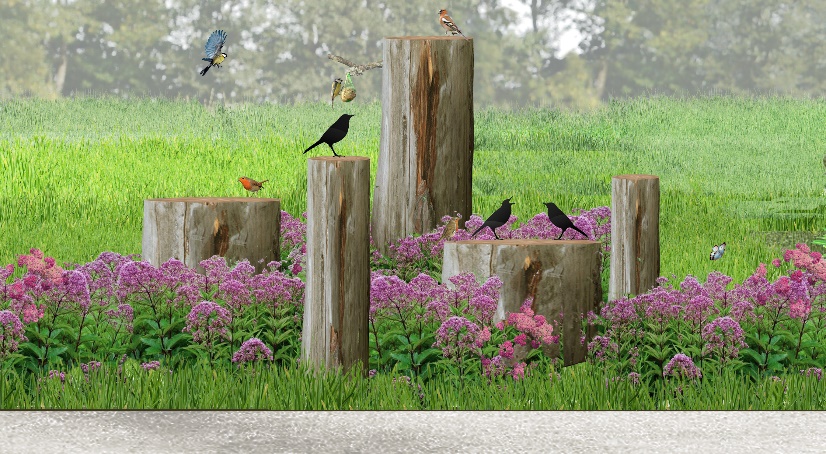


Figure 5 The bird garden


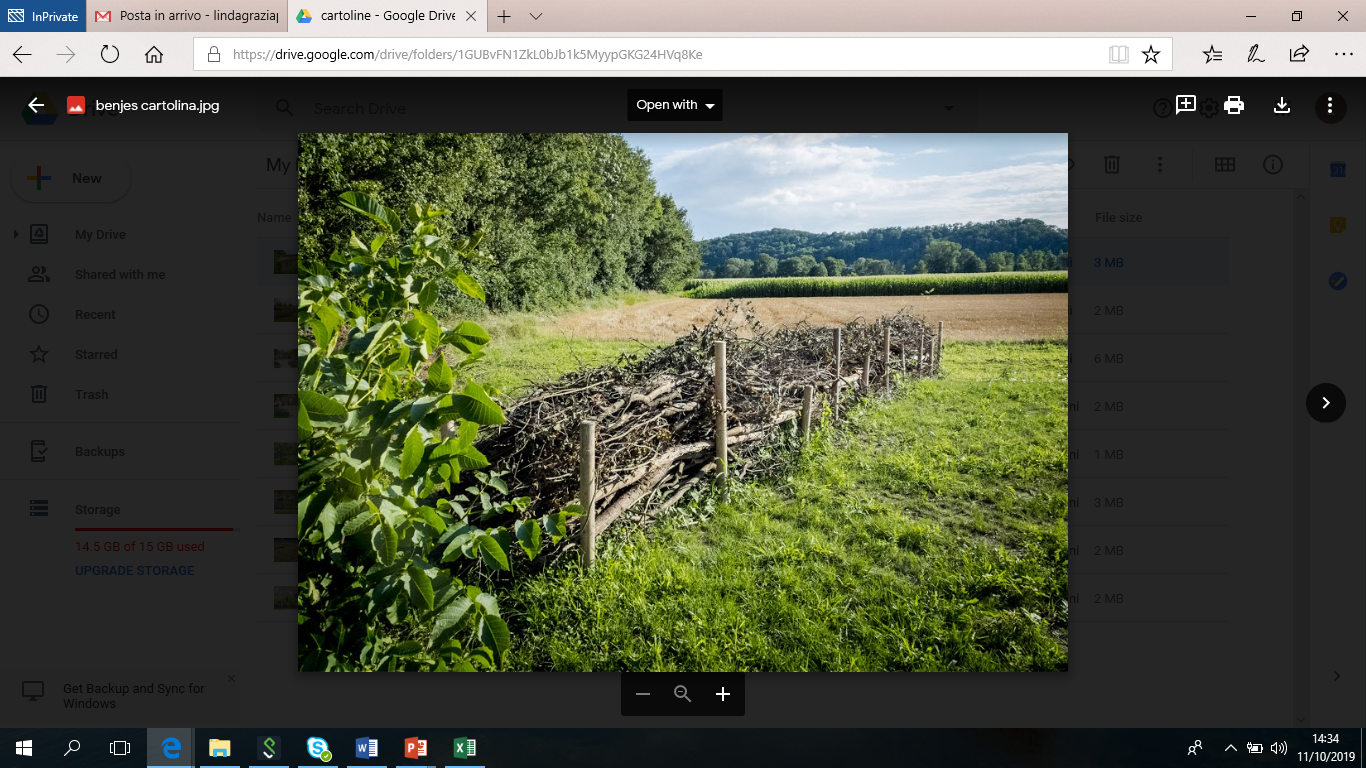


Figure 6 The Benje’s hedge


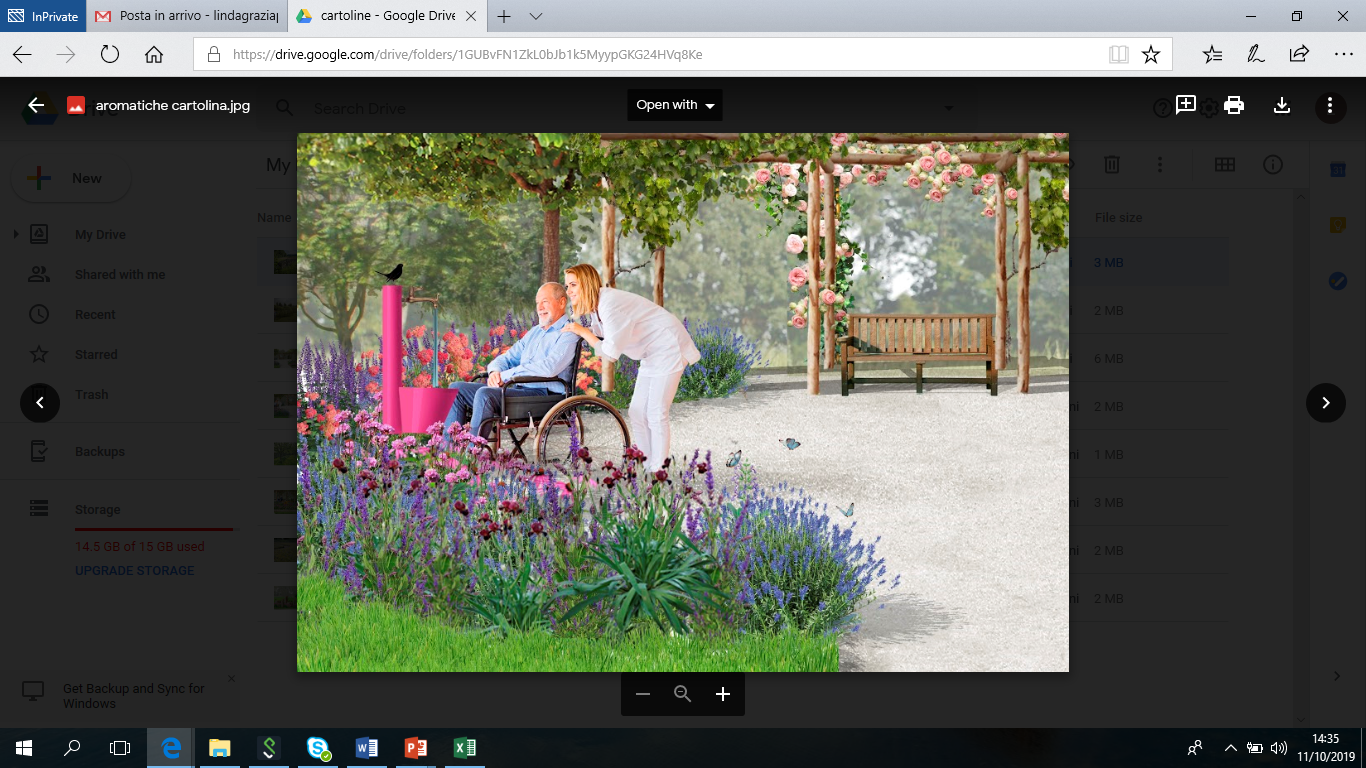


Figure 7 The drinking fountain
